# Association between metabolic dysfunction-associated steatotic liver disease and risk of incident pancreatic cancer: a systematic review and meta-analysis of cohort studies

**DOI:** 10.3389/fonc.2024.1366195

**Published:** 2024-03-19

**Authors:** Yi Zhang, Ben-Gang Zhou, Ji-Dong Zhan, Bin-Bin Du

**Affiliations:** ^1^ Department of General Medicine, The Hospital of Huazhong University of Science and Technology, Wuhan, Hubei, China; ^2^ Dalian Medical University, Dalian, Liaoning, China

**Keywords:** non-alcoholic fatty liver disease, metabolic dysfunction-associated steatotic liver disease, pancreatic cancer, cohort studies, meta-analysis, systematic review

## Abstract

**Background and objectives:**

Since the results of previous observational studies on the relationship between metabolic dysfunction-associated steatotic liver disease (MASLD) and pancreatic cancer were still controversial and inconsistent, we performed a systematic evaluation and meta-analysis of cohort studies to assess any potential association.

**Methods:**

We conducted a systematic search of PubMed, Embase, and Web of Science databases from the database’s inception up to November 30, 2023. For summary purposes, hazard ratios (HRs) with 95% confidence intervals (CIs) were calculated using random-effects models, and subgroup and sensitivity analyses were performed as well. The Egger’s test and Begg’s test were utilized to detect the publication bias.

**Results:**

This meta-analysis included nine cohort studies with a total of 10,428,926 participants. The meta-analysis demonstrated an increased risk of pancreatic cancer in those with MASLD (HR = 1.32, 95% CI: 1.10-1.59, *P* = 0.003) with moderate heterogeneity (I^2 ^= 54%, *P* = 0.03). Subsequent subgroup analyses revealed that the pooled HRs remained significantly unchanged, irrespective of the study area, nomenclature of fatty liver disease, and sample size. The results of the sensitivity analyses remained unchanged. No evidence of publication bias was found.

**Conclusion:**

This meta-analysis indicated that MASLD was associated with a higher risk of pancreatic cancer. To further strengthen the association, future prospective cohort studies should take into account different ethnic groups, diagnostic methods of fatty liver, the severity of MASLD, and potential confounding factors, as well as explore the potential mechanisms of pancreatic cancer development in MASLD patients.

**Systematic review registration:**

https://www.crd.york.ac.uk/PROSPERO/, identifier: CRD42023489137.

## 1 Introduction

Metabolic dysfunction-associated steatotic liver disease (MASLD), formerly known as non-alcoholic fatty liver disease (NAFLD) or metabolic dysfunction-associated fatty liver disease (MAFLD) (1–3), has become the most widespread form of chronic liver disease, with an estimated 30% of adults worldwide suffering from it (4). It is also highly prevalent in children and adolescents, with an estimated 7.4% of them having it (5). Recent research conducted with the American population indicated that alterations to diagnostic criteria (from NAFLD to MASLD) do not result in a notable change in disease prevalence (6). It is estimated that one in five people with MASLD will experience metabolic dysfunction-associated steatohepatitis (MASH), which is the main cause of cirrhosis and hepatocellular carcinoma (7). Liver cancer is the second leading cause of mortality from all cancers (8). Progressive MASLD or MASH can cause liver death and is linked to a decline in health-related quality of life, a decrease in worker productivity, and an increase in medical resource utilization, leading to greater medical costs and economic burden (7, 9). Over the last few years, mounting evidence has suggested that MASLD is a complex illness with consequences beyond the liver, including MASH, cirrhosis, or liver cancer, as well as possible connections to diabetes, chronic kidney disease, cardiovascular disease, and extrahepatic cancer (10–14).

Pancreatic cancer is an extremely deadly disease, with a 5-year survival rate of only about 10% in the USA (15). It is the seventh most common cause of cancer-related deaths for both males and females globally (16). For the majority of patients, ranging from 80 to 85%, they experience either unresectable or metastatic diseases. Even with tumors that can be removed locally, the 5-year survival rate is still only 20% (15). However, the cause of pancreatic cancer is still a mystery. Determining the risk factors of pancreatic cancer and taking preventive action based on those risks are of great importance for public health.

In recent years, there has been a surge in curiosity about the relationship between MASLD and pancreatic cancer. Previously, there were two meta-analyses (17, 18) explored the association between MASLD and multiple extrahepatic cancers. In the two meta-analyses, only three studies (published between 2015 and 2019) were included to explore the association between MASLD and pancreatic cancer. The results of the two meta-analyses both indicated that MASLD patients had an increased risk of pancreatic cancer. However, the small number of included studies and case control study designs used in the research weakened the accuracy and reliability of the results. Additionally, in the past three years, a large number of high-quality cohort studies on this topic have been published, but the results were still inconsistent. These new studies have sparked our interest in updating the existing evidence.

Based on the above considerations, we conducted a systematic review and meta-analysis of cohort studies to comprehensively and accurately assess the relationship between NAFLD/MAFLD and pancreatic cancer risk. This will provide a reference for better prevention of pancreatic cancer in clinical practice.

## 2 Materials and methods

This meta-analysis was registered in advance with the PROSPERO platform (https://www.crd.york.ac.uk/PROSPERO/, registration number: CRD42023489137). We followed the PRISMA statement (19) and MOOSE reporting guidelines (20) while carrying out this study.

### 2.1 Search strategy

We conducted a systematic search of PubMed, Embase, and Web of Science databases without language limitations, with studies published from the database’s inception up to November 30, 2023. The following search strategy was used to search PubMed for information related to MASLD and pancreatic cancer: (“non-alcoholic fatty liver disease”[MeSH Terms] OR “non-alcoholic fatty liver disease”[All Fields] OR “nonalcoholic fatty liver disease”[All Fields] OR “non-alcoholic fatty liver”[All Fields] OR “nonalcoholic fatty liver”[All Fields] OR “nonalcoholic steatohepatitis”[All Fields] OR “non-alcoholic steatohepatitis”[All Fields] OR “fatty liver”[MeSH Terms] OR “fatty liver”[All Fields] OR “Metabolic dysfunction-associated fatty liver disease”[All Fields] OR “Metabolic associated fatty liver disease”[All Fields] OR “Metabolic Dysfunction-Associated Steatotic Liver Disease”[All Fields] OR NAFLD[All Fields] OR NASH[All Fields] OR NAFL[All Fields] OR MAFLD[All Fields] OR MASLD[All Fields]) AND [(“pancreas”[MeSH Terms] OR “pancreas”[All Fields]) OR (“pancreatic”[All Fields])] AND [(“cancer”[All Fields] OR “cancers”[All Fields]) OR (“tumor”[All Fields] OR “tumour”[All Fields]) OR (“tumors”[All Fields] OR “tumours”[All Fields]) OR (“neoplasms”[MeSH Terms] OR “neoplasms”[All Fields] OR “neoplasm”[All Fields]) OR (“carcinoma”[MeSH Terms] OR “carcinoma”[All Fields]) OR (“adenocarcinoma”[MeSH Terms] OR “adenocarcinoma”[All Fields])]. This search strategy was modified to suit the Embase and Web of Science databases. To guarantee a comprehensive search, references of all applicable original studies and review articles were also scrutinized to locate extra studies.

### 2.2 Study selection

The criteria for inclusion in this study were the following (1): Cohort studies investigating the association between MASLD and risk of pancreatic cancer (2); The diagnosis of MASLD was based on the previous nomenclature of NAFLD/MAFLD, NAFLD was determined by ultrasonography (USG), International Classification of Diseases (ICD) codes, fatty liver index (FLI), or liver biopsy, when excessive alcohol use and other causes of liver disease were ruled out. MAFLD was identified through imaging techniques, histological (liver biopsy) or blood biomarker evidence of fat accumulation in the liver (hepatic steatosis) combined with one of the following three criteria: overweight/obesity, type 2 diabetes mellitus (T2DM), or evidence of metabolic dysregulation (3) (3); the confirmation methods of pancreatic cancer were based on ICD codes, medical records, or pathological and/or imaging techniques (4); Studies that report hazard ratios (HRs), risk ratios (RRs), or incidence rate ratios (IRRs) with 95% confidence intervals (CIs) values for the outcome of interest, or studies that provide raw data to calculate them (5); In cases of overlapping populations, the study with the largest sample size was chosen for inclusion. Excluded from the criteria were cross-sectional or case-control studies, conference abstracts, editorials, letters, comments, reviews and meta-analyses, duplicate publications, and studies without relevant data and an appropriate control group. Both investigators independently selected all the eligible studies based on the criteria, and any discrepancies were solved through discussion.

### 2.3 Data extraction

The two investigators extracted data from each selected study and appraised the methodology, resolving any disagreements by consensus. The extracted data included the surname of the first author, publication year, study country, study design, source of study subjects, sample size, participants characteristics, diagnostic methods of fatty liver and pancreatic cancer, follow-up time, HRs, RRs, or IRRs with their 95% CI, and adjusted confounding factors.

### 2.4 Quality assessment

The Newcastle-Ottawa Scale (NOS) (21) for cohort studies was employed to carry out a methodological quality assessment. The scale evaluates a study with a star system of up to 9 stars, covering three domains: selection of participants (up to four stars), comparability of study groups (up to two stars), and ascertainment of outcomes of interest (up to three stars). We classified studies with nine stars as high quality, those with seven or eight stars as moderate quality, and those with six or fewer stars as low quality (11).

### 2.5 Statistical analysis

The Review Manager 5.3 software (The Cochrane Collaboration, Copenhagen, Denmark) was utilized for conducting meta-analyses. The DerSimonian and Laird generic inverse variance method, based on a random-effects model, was used to estimate the effect size (22). Given the relatively low incidence of outcome of interest, RRs/IRRs were approximated by HRs. The effect size of each eligible study was determined by the HRs with 95% CIs. When encountering adjusted HRs/RRs/IRRs in a report, the one with the most confounders was preferred. We evaluated the differences between studies by means of the Cochran’s Q-test (with a *p*-value of < 0.10) and the I² statistic. I^2^-values around 25% indicate low heterogeneity, while values around 50% indicate medium heterogeneity, and values around 75% indicate high heterogeneity (23). To examine the effect of particular study and participant characteristics on the results and identify potential sources of heterogeneity, we carried out numerous subgroup analyses according to study area, nomenclature of FLD, sample size, follow-up time and diagnostic methods of fatty liver. Moreover, we conducted sensitivity analysis by excluding individual studies one by one to assess the possible excessive influence of individual studies on the overall pooled estimates. To evaluate the potential publication bias, Begg’s funnel plot, Egger’s test (24) and Begg’s test (25) were inspected using STATA/SE 12.0 software (STATA Corporation, Texas, USA). Statistical significance was determined at *P* < 0.05 (*P* < 0.10 for the Cochran’s Q- test).

## 3 Results

### 3.1 Study selection process

**Figure 1:**
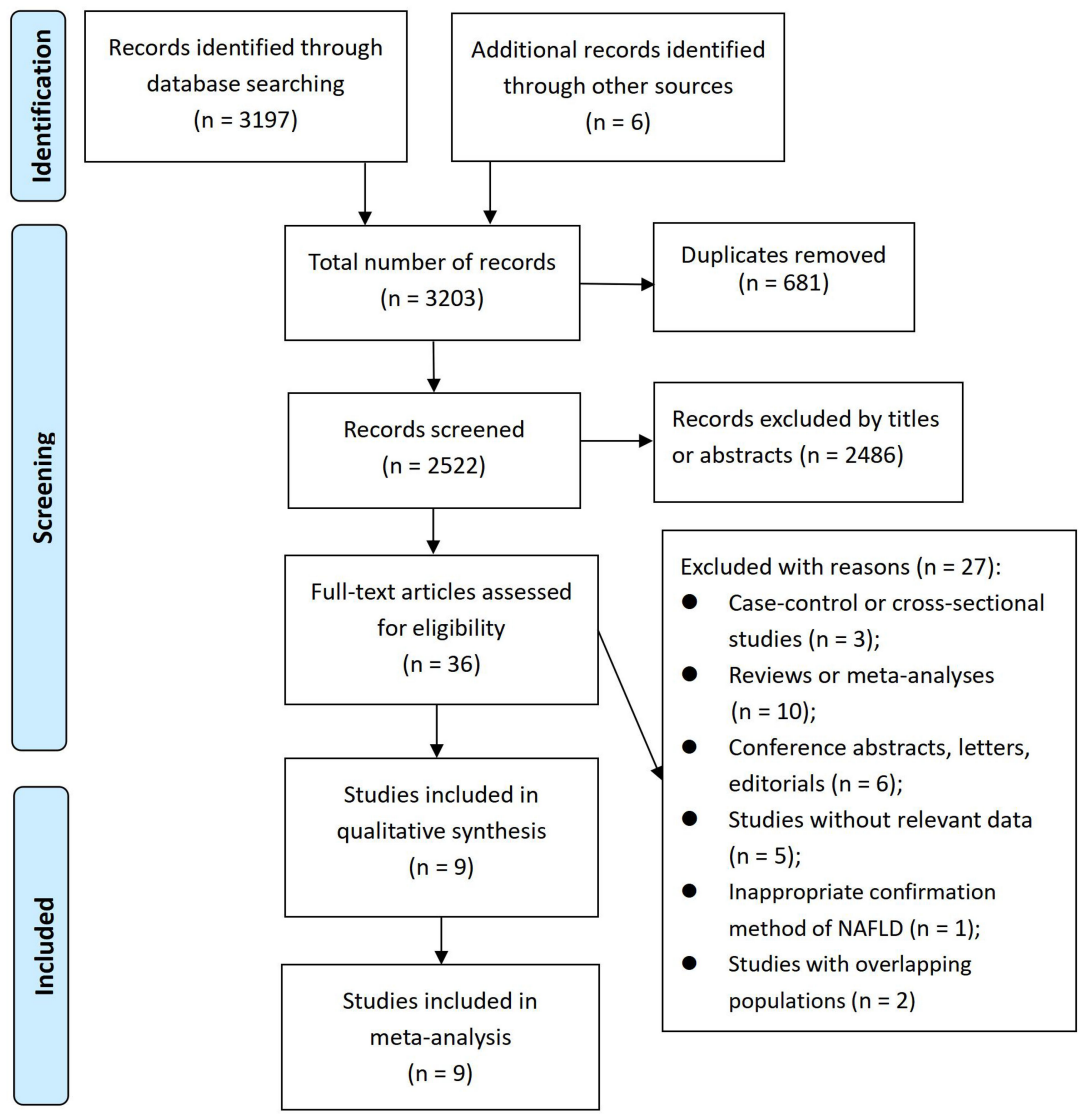
PRISMA flowchart of study selection process.

A total of 3203 records were identified. After deleting 681 duplicate records from the title, further 2486 records were removed based on their relevance from the title and abstract. Subsequently, 36 articles underwent a full-text evaluation, out of which 27 articles were excluded for various reasons (see **Figure 1** and **Supplementary Table S1**). As a result, nine cohort studies (26–34) were included in our meta-analysis.

### 3.2 Study characteristics and quality assessment

The main characteristics of the studies included can be found in **Tables 1** and **Supplementary Table S2**. The meta-analysis consisted of nine cohort studies between 2015 and 2023, involving a total of 10,428,926 participants. Out of the nine studies, four examined the connection between MAFLD (the previous nomenclature of MASLD) and pancreatic cancer (31–34). The research was conducted in countries from three continents. In Asia, there were six studies (26, 27, 30, 32–34) from South Korea and China, while in Europe, there were two studies (29, 31) from Sweden and the United Kingdom, and in North America, there was one study (28) from the United States. The number of people in the studies varied from 10,545 to 9,718,182. For the diagnosis of fatty liver, two studies (26, 28) used ICD codes, two studies (31, 32) utilized FLI, four studies (27, 30, 33, 34) employed USG, and only one study (29) relied on liver biopsy. Most studies for confirming pancreatic cancer utilize ICD codes. The average length of the follow-up period was between 3.3 and 13.8 years. All studies revealed that confounders were taken into account (**Supplementary Table S2**). Four studies (29, 31, 32, 34) with a NOS score of 9 stars were considered to be of high quality, while five studies (26–28, 30, 33) with a NOS score of 7 or 8 stars were judged to be of medium quality in terms of methodological quality assessment (see **Supplementary Table S3** for further details).

**Table 1:** Main characteristics of included studies.

Study (Year)	Country	Study period	Sample size	Diagnosis of fatty liver	Confirmation of pancreatic cancer	Follow-up time (mean years)	HR/IRR (95%CI)	NOS score
Sun (2015) (26)	China	2000-2011	10,545	ICD codes	ICD codes	3.6	HR 2.72 (0.93-7.95)	8
Kim (2018) (27)	South Korea	2004-2005	25,947	USG	Pathological and/or radiological criteria	7.5	IRR 1.16 (0.51-2.65)	7
Allen (2019) (28)	USA	1997-2016	19,163	ICD codes	ICD codes	8	IRR 2.0 (1.2-3.3)	7
Simon (2021) (29)	Sweden	1966-2016	48,799	Liver biopsy	Pathological and/or radiological criteria	13.8	HR 2.15 (1.40-3.30)	9
Wang (2021) (30)	China	2006-2007	54,187	USG	ICD codes	10	HR 0.87 (0.50-1.52)	8
Liu (2022) (31)	UK	2002-2010	352,911	FLI	ICD codes	8.2	HR 1.31 (1.10-1.56)	9
Chung (2023) (32)	South Korea	2009.1-2009.12	9,718,182	FLI	ICD codes	8.3	HR 1.16 (1.04-1.29)	9
Wei (2023) (33)	China	2013-2021	47,801	USG	ICD codes	3.3	HR 1.87 (0.59-5.95)	8
Yuan (2023) (34)	China	2006-2014	151,391	USG	Medical records	12.6	HR 0.97 (0.68-1.38)	9

HR, hazard ratio; IRR, incidence rate ratio; CI, confidence interval; NOS, Newcastle-Ottawa Scale; ICD, international classification of diseases; USG, Ultrasonography; FLI, Fatty liver index; USA, United States; UK, United Kingdom.

### 3.3 Association between MASLD and pancreatic cancer

#### 3.3.1 Overall meta-analysis

**Figure 2:**
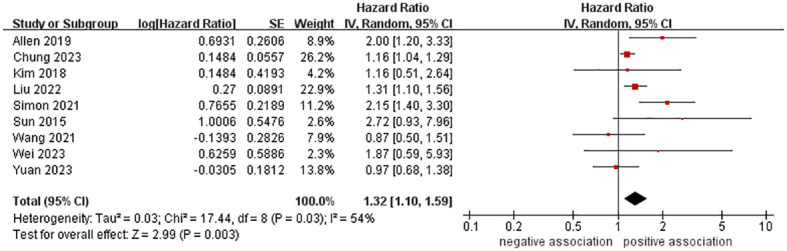
Forest plot of overall meta-analysis of association between MASLD and risk of pancreatic cancer.

A total of 10,428,926 participants were included in nine cohort studies to examine the relationship between MASLD and the risk of pancreatic cancer. The meta-analysis demonstrated an increased risk of pancreatic cancer in those with MASLD (HR = 1.32, 95% CI: 1.10-1.59, *P* = 0.003). The pooled analysis showed moderate heterogeneity (I^2 ^= 54%, *P* = 0.03) (**Figure 2**).

#### 3.3.2 Subgroup and sensitivity analyses

**Figure 3:**
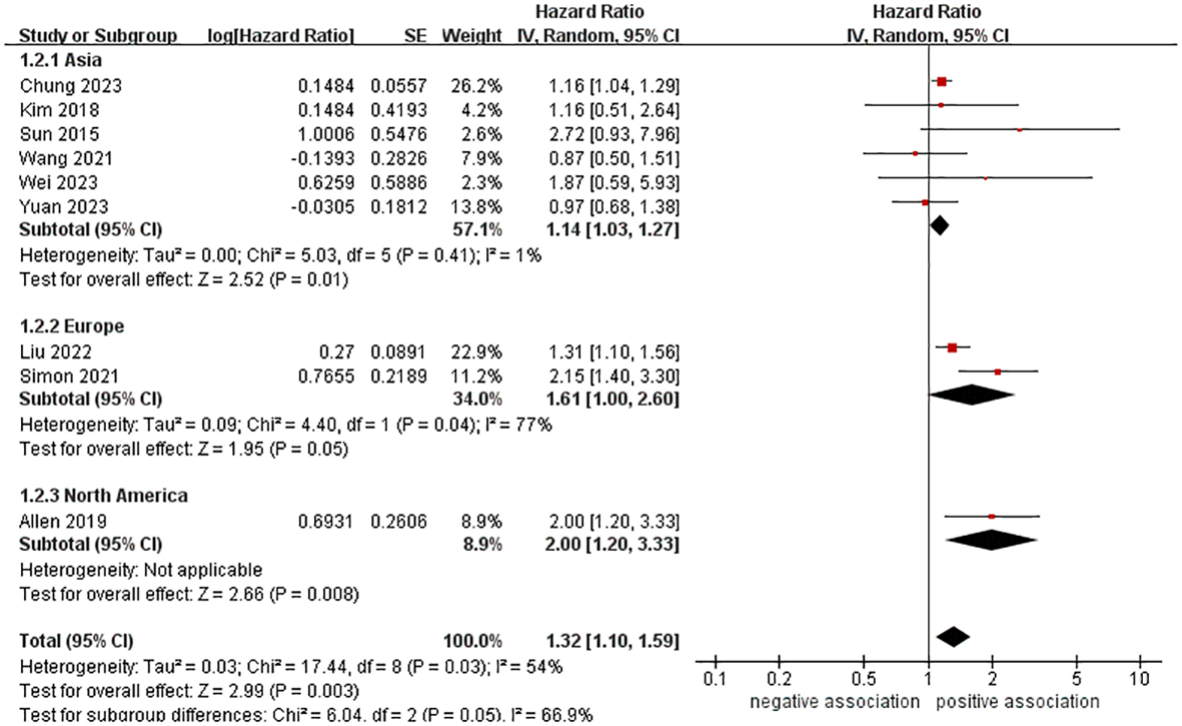
Forest plot of subgroup analysis based on study areas.

**Figure 4:**
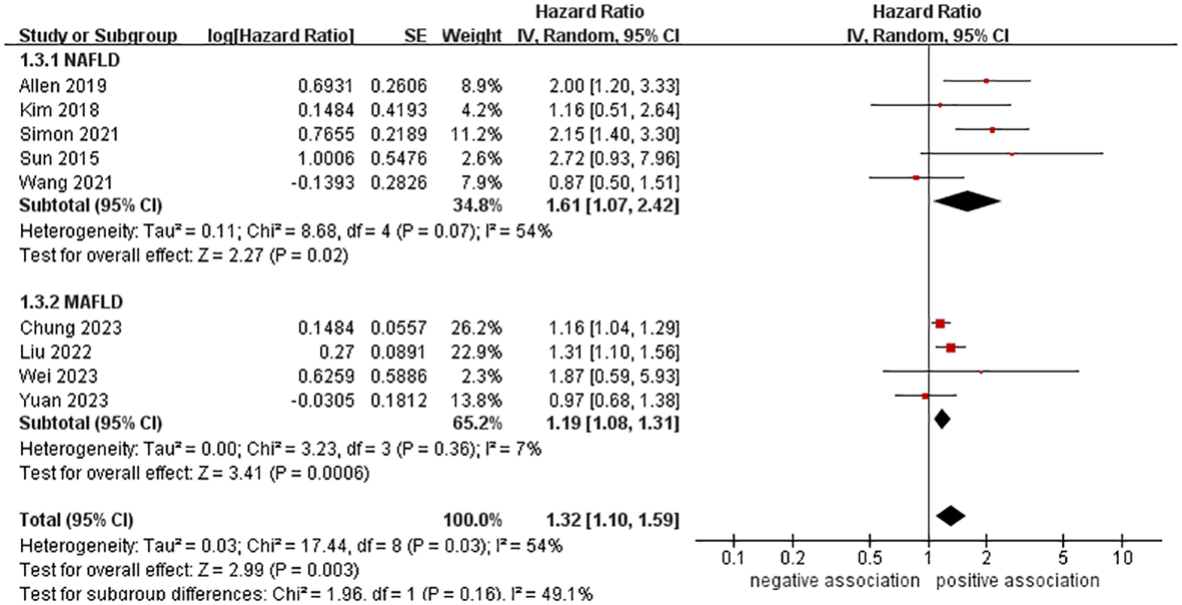
Forest plot of subgroup analysis based on nomenclature of fatty liver disease.

**Figure 5:**
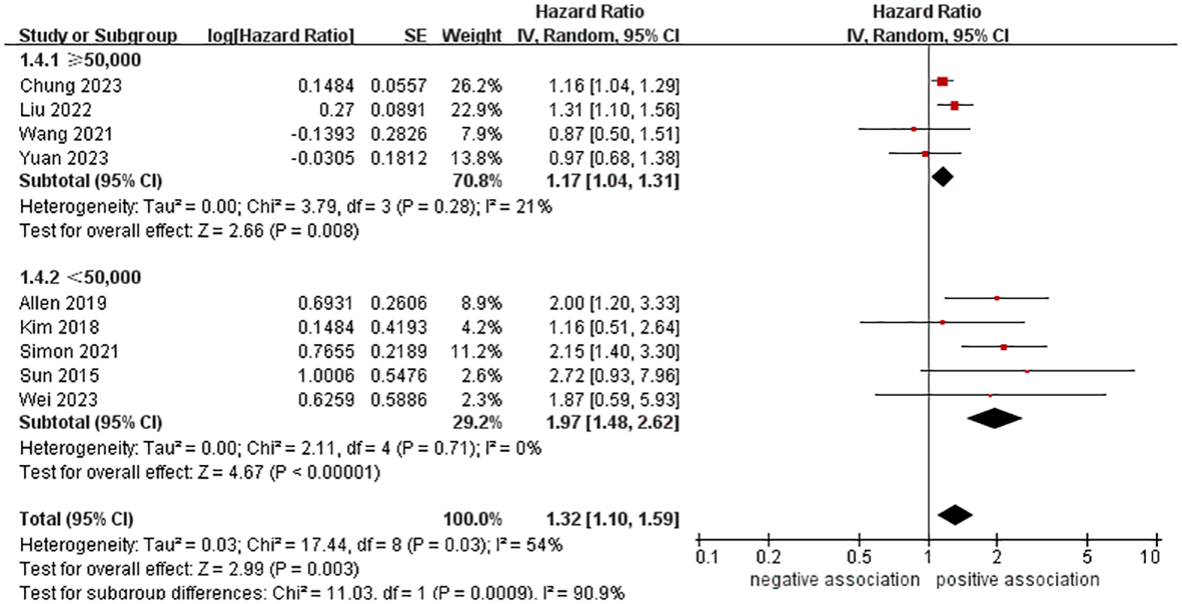
Forest plot of subgroup analysis based on sample size.

**Figure 6:**
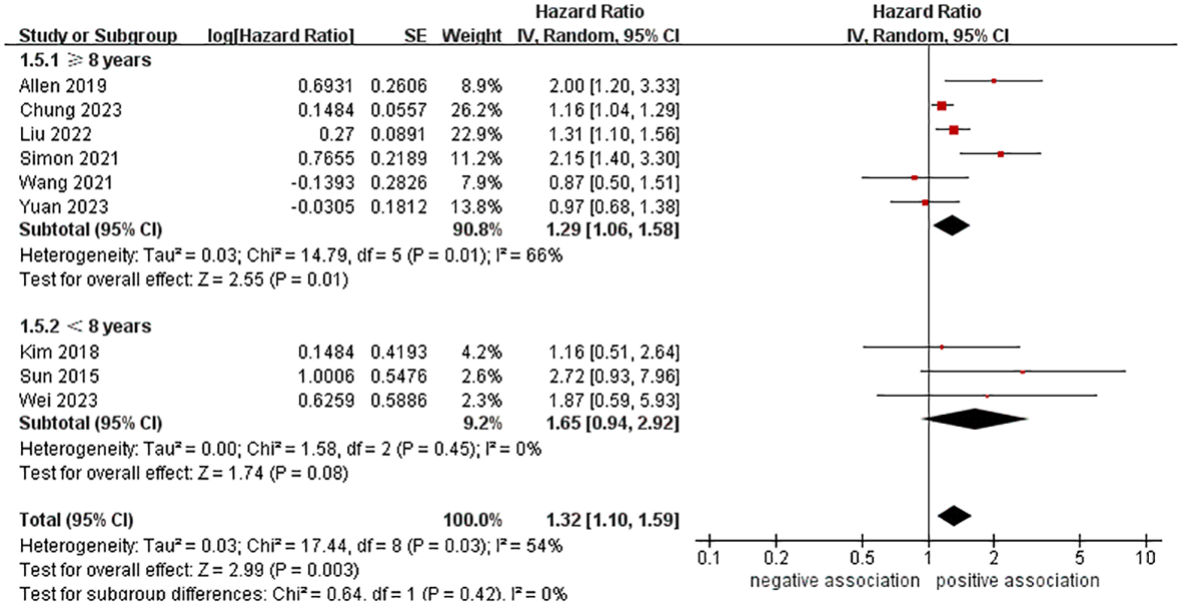
Forest plot of subgroup analysis based on follow-up time.

**Figure 7:**
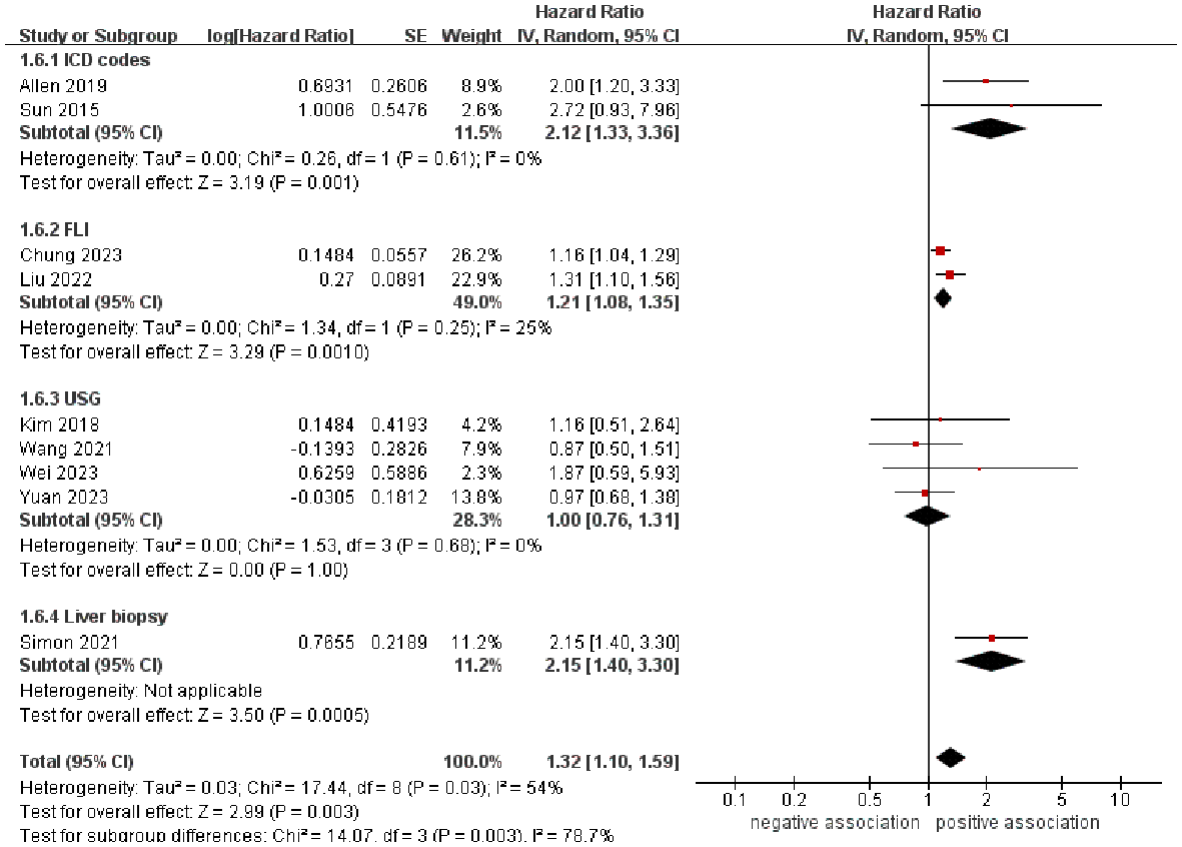
Forest plot of subgroup analysis based on the diagnosis of fatty liver.

In order to explore potential factors impacting the general results and the potential sources of heterogeneity among the studies, we performed multiple subgroup analyses. The results of the subgroup analyses based on the study area, nomenclature of FLD, and sample size were in line with the overall summary results (**Figures 3**–**5**). When we conducted subgroup analysis based on follow-up time, we discovered that MASLD was correlated with an increased risk of pancreatic cancer in the subgroup with follow-up time equal to or exceeding 8 years (n = 6, HR = 1.29, 95% CI: 1.06-1.58, *P* = 0.01), but there was no significant statistical difference between the two in the subgroup with follow-up time less than 8 years (n = 3, HR = 1.65, 95% CI: 0.94-2.92, *P* = 0.08) (**Figure 6**). In the subgroup analysis based on the diagnosis of fatty liver, we discovered that when using ICD codes (n = 2, HR = 2.12, 95% CI: 1.33-3.36, *P* = 0.001), FLI (n = 2, HR = 1.21, 95% CI: 1.08-1.35, *P* = 0.001) and liver biopsy (n = 1, HR = 2.15, 95% CI: 1.40-3.30, *P* = 0.0005) for diagnosis, MASLD is linked to a heightened risk of pancreatic cancer. However, when USG was utilized to diagnose MASLD, there is no such correlation between MASLD and risk of pancreatic cancer (n = 4, HR = 1.00, 95% CI: 0.76-1.31, *P* = 1.00) (**Figure 7**). It is noteworthy that when we conducted subgroup analyses based on the sample size and diagnosis of fatty liver, the I^2^ of each subgroup decreased to a certain extent, with the majority decreasing to 0. This indicated that the sample size and diagnostic method of fatty liver were sources of heterogeneity. The results of subgroup analyses are shown in **Table 2**.

**Table 2:** The results of subgroup analyses.

Subgroups	No. of studies	HR (95%CI)	*P*_association_	*I^2^* (%)	*P*_heterogeneity_
Study areas					
Asia	6	1.14 (1.03-1.27)	0.01	1	0.41
Europe	2	1.61 (1.00-2.60)	0.05	77	0.04
North America	1	2.00 (1.20-3.33)	0.008	-	-
Nomenclature of FLD					
NAFLD	5	1.61 (1.07-2.42)	0.02	54	0.07
MAFLD	4	1.19 (1.08-1.31)	0.0006	7	0.36
Sample size					
≥50,000	4	1.17 (1.04-1.31)	0.008	21	0.28
<50,000	5	1.97 (1.48-2.62)	< 0.00001	0	0.71
Follow-up time					
≥ 8 years	6	1.29 (1.06-1.58)	0.01	66	0.01
< 8 years	3	1.65 (0.94-2.92)	0.08	0	0.45
Diagnosis of fatty liver					
ICD codes	2	2.12 (1.33-3.36)	0.001	0	0.61
FLI	2	1.21 (1.08-1.35)	0.001	25	0.25
USG	4	1.00 (0.76-1.31)	1.00	0	0.68
Liver biopsy	1	2.15 (1.40-3.30)	0.0005	-	-
**Overall studies**	**9**	**1.32 (1.10-1.59)**	**0.003**	**54**	**0.03**

FLD, fatty liver disease; NAFLD, nonalcoholic fatty liver disease; MAFLD, Metabolic dysfunction-associated fatty liver disease; HR, hazard ratio; CI, confidence interval; ICD, International Classification of Diseases; FLI, Fatty liver index; USG, Ultrasonography.

Bold values indicate the results of overall meta-analysis.

To ensure the reliability of the results, sensitivity analysis was performed by removing individual studies one by one and the results remained consistent, indicating that the results were stable (see **Table 3**).

**Table 3:** Results of sensitivity analyses .

Studies omitted	HR (95% CI)	*P*_association_	Heterogeneity
Sun (2015) (26)	1.29 (1.08-1.55)	0.005	*I* ^2 ^= 54%, *P* = 0.03
Kim (2018) (27)	1.34 (1.10-1.62)	0.003	*I* ^2 ^= 60%, *P* = 0.01
Allen (2019) (28)	1.26 (1.06-1.51)	0.010	*I* ^2 ^= 49%, *P* = 0.05
Simon (2021) (29)	1.23 (1.06-1.43)	0.008	*I* ^2 ^= 34%, *P* = 0.15
Wang (2021) (30)	1.37 (1.13-1.66)	0.001	*I* ^2 ^= 56%, *P* = 0.03
Liu (2022) (31)	1.36 (1.05-1.76)	0.02	*I* ^2 ^= 58%, *P* = 0.02
Chung (2023) (32)	1.40 (1.09-1.80)	0.008	*I* ^2 ^= 52%, *P* = 0.04
Wei (2023) (33)	1.31 (1.09-1.59)	0.005	*I* ^2 ^= 59%, *P* = 0.02
Yuan (2023) (34)	1.39 (1.14-1.70)	0.001	*I* ^2 ^= 55%, *P* = 0.03

#### 3.3.3 Publication bias assessment

Examination of the Begg’s funnel plot revealed a slightly asymmetrical distribution (**Supplementary Figure S1**). Nevertheless, no significant publication bias was evident, as the Begg’s test and Egger’s test both indicated (*P*_Begg_= 0.466, *P*_Egger_= 0.242).

### 3.4 Association between the severity of MASLD and pancreatic cancer

Only one study (26) explored the relationship between the severity of MASLD (cirrhosis) and pancreatic cancer. The results showed that there was no significant association between MASLD-related cirrhosis and the increase of pancreatic cancer (HR=2.72, 95% CI: 0.93-7.95).

## 4 Discussion

### 4.1 Main findings of our meta-analysis

This present meta-analysis pooled all available data (nine cohort studies with 10,428,926 participants) to quantify the association between MASLD and pancreatic cancer risk. We found that MASLD was linked with an increased probability of developing pancreatic cancer (HR = 1.32, 95% CI: 1.10-1.59, *P* = 0.003). Subsequent subgroup analyses revealed that the magnitude of the risk remained significantly unchanged, irrespective of the study area, nomenclature of FLD, and sample size. The results were further validated by sensitivity analysis.

### 4.2 Comparison with previous work

To our knowledge, this is the latest and largest meta-analysis to examine the relationship between MASLD and the risk of pancreatic cancer separately, only considering cohort studies. A previous meta-analysis conducted by Liu et al. (17) explored the relationship between MASLD and extrahepatic cancers. In their meta-analysis, only three observational studies (two cohort studies and one case-control study) on the relationship between MASLD and pancreatic cancer were included. The results showed that MASLD patients had an increased risk of pancreatic cancer (OR=2.12, 95% CI: 1.58-2.83). Our meta-analysis included two cohort studies that had been a part of the prior meta-analysis, with the exception of one case-control study that could be more prone to bias. In 2022, Mantovani et al. (18) conducted a similar meta-analysis to investigate the association between MASLD and incident of extrahepatic cancers, which included only three cohort studies related to MASLD and pancreatic cancer. The results showed that MASLD could increase the risk of pancreatic cancer (HR=1.84, 95% CI 1.23-2.74).

Compared to previous smaller meta-analyses, our updated meta-analysis confirms and further expands past work. Firstly, our meta-analysis included all cohort studies that were featured in previous meta-analyses, as well as the eight most recent additional cohort studies published between 2021 and 2023. This provided the most up-to-date, largest, and comprehensive epidemiological evidence related to this topic. Secondly, we avoided case-control studies, which are more prone to recall bias, and instead included only medium to high-quality cohort studies in our meta-analysis. This ensured that our results were more reliable. Thirdly, we conducted a more thorough analyses, including multiple subgroup analyses and sensitivity analyses, further validating the reliability and stability of the results. Finally, by including four studies that examine the relationship between MAFLD (the novel terminology of NAFLD) and pancreatic cancer, which were not present in the previous meta-analyses, we further strengthen the evidence on this topic.

### 4.3 Potential explanations and implications

Pancreatic cancer is significantly associated with obesity, a well-established risk factor (35). MASLD is the hepatic manifestation of metabolic syndrome (2). Recent research indicates that weight loss surgery can provide a protective benefit against pancreatic cancer in individuals between the ages of 18 and 50 (35). It remains uncertain, from a pathophysiological perspective, whether MASLD is an independent risk factor for pancreatic cancer or if it is solely linked to the elevated risk of pancreatic cancer resulting from common metabolic risk factors such as obesity. There were several potential pathophysiological mechanisms in recent research. Firstly, inflammatory responses may be a vital component in the connection between MASLD and pancreatic cancer. MASLD is characterized by a low-grade systemic inflammation (36). Chronic inflammation has been found to be related to many malignancies and is the main cause of many malignant tumors (37, 38). Adiposity and its associated chronic inflammation lead to an increase in the secretion of tumor necrosis factor alpha (TNF-α) and leptin, and a decrease in the secretion of adiponectin. This inflammation and disruption of adipocytokine signaling worsens insulin resistance (IR), and can also stimulate cell proliferation, tumor progression, and cancer angiogenesis (39). Secondly, IR is a critical element in the etiology of MASLD (40). IR causes chronic inflammation, which then activates the insulin-like growth factor 1 (IGF-1) axis, thus creating a conducive environment for cancer growth. IR causes chronic hyperinsulinemia, which in turn reduces the production and release of IGF-binding protein 1 and 2 by the liver. This leads to higher levels of bioavailable IGF-1, which, together with insulin, can speed up the accumulation of mutations, stimulate cell growth, and prevent cell death in many tissues, thus encouraging the development of cancer (39). Thirdly, Intestinal dysbiosis may be a significant factor in the cause of MASLD and the emergence of pancreatic cancer (41, 42). Dysbiosis of the intestines can cause an increase in intestinal permeability, allowing bacterial products to enter the body’s circulation, activating Toll like receptors (TLRs) in the process. This recognition of microbial-related molecules can lead to carcinogenesis (14). In addition, carcinogenesis can be induced by bacterial metabolites such as secondary bile acids, polyamines, hydrogen sulfide, and reactive oxygen species, which can cause DNA damage and inflammation through the production of TNF-αand interleukin (IL)-6 (39). Finally, diabetes and hyperglycemia are both potential risk factors for cancer, and they are the determinants of MASLD. MASLD is a combination of diabetes and hyperglycemia, and high blood sugar levels can lead to oxidative stress and damage to DNA due to increased oxidation of mitochondrial glucose (32, 43, 44).

To address the potential influence of different diagnostic methods of fatty liver on the outcomes, subgroup analysis was carried out according to the specific diagnostic methods. The meta-analysis revealed that there was no statistically significant association between MASLD and the increased risk of pancreatic cancer when fatty liver diagnosis was based on USG. One potential reason could be that the limited number of studies and small sample sizes lead to a lack of statistical testing efficiency, thereby hindering the ability to make significant conclusions. Additionally, USG is currently the primary imaging technique used to diagnose hepatic steatosis, demonstrating high sensitivity and specificity in detecting moderate steatosis. However, its accuracy decreases when steatotic hepatocytes are less than 10%-12.5% (45–47). Consequently, using the USG diagnostic method may result in some patients with mild fatty liver being disregarded.

Given the high prevalence and severe burden of MASLD, we believe that our meta-analysis holds great significance for clinical practice. Our research has shown that MASLD should not be overlooked. Physicians should be aware of the potential risks associated with these conditions and track patients accordingly to detect pancreatic cancer in its early stages. To accurately evaluate the risk of pancreatic cancer in the population due to MASLD, further prospective large cohorts are needed to explore the causality relationship between MASLD and pancreatic cancer, taking into account factors such as race, diagnosis methods, severity of the condition, and potential confounding variables like smoking, drinking, obesity, and pancreatitis. Clinicians should be alert to the possibility of cancer in these individuals and conduct screenings when necessary.

### 4.4 Strengths and limitations

This present meta-analysis has several strengths. This thorough systematic review and meta-analysis, conducted with a meticulous approach, has established the connection between MASLD and risk of pancreatic cancer. Subgroup analyses and sensitivity analyses were utilized to assess the dependability of the combined risk estimation. Moreover, all the cohort studies included had a medium to high quality, and there was no significant publication bias observed, thereby guaranteeing the validity of the research results.

Despite the above strengths of our meta-analysis, a few potential limitations should be taken into account. Firstly, although all included studies attempted to account for confounding factors, the exact confounding factors adjusted for varied, and the majority of the eligible studies did not make any adjustments, or only made partial adjustments, for risk factors like smoking, drinking, family history of pancreatic cancer, chronic pancreatitis, and diabetes. Moreover, residual and unmeasured confounding factors cannot be ruled out, which may affect the estimation of the results. Secondly, our meta-analysis revealed a moderate level of heterogeneity (I^2^ = 54%), however, we conducted subgroup analysis and discovered that the sample size and diagnostic methods of fatty liver were the sources of heterogeneity. The results of these subgroup analyses were similar to the overall results. Thirdly, owing to the data limitations provided by the included studies, we cannot analyze further the severity of MASLD (such as MASH, liver fibrosis or cirrhosis) and the risk of pancreatic cancer. Subsequent studies should delve further into this issue. Fourthly, due to the lack of relevant information on the treatment of MASLD in the original studies included, we cannot further evaluate its impact on the results. Fifthly, the small number of some subgroups may lead to insufficient statistical efficiency and the inability to draw meaningful conclusions. It is imperative that more high-quality research is conducted in the future to address this issue effectively. Sixthly, the duration of follow-up time included in the study might have an impact on the results. Our subgroup analysis based on follow-up time revealed that the increased risk of MASLD and pancreatic cancer was more evident in the subgroup with a follow-up time of 8 years or more. However, there was no significant association between MASLD and an increased risk of pancreatic cancer in the subgroup with a follow-up time of less than 8 years. This could be due to either fewer studies being included in subgroups with shorter follow-up times or the outcomes not being observed within a short follow-up period. Finally, the majority of the studies included were from Asia, while there is a lack of research evidence from Europe and North America. Body fat distribution, lifestyle habits, and genetic backgrounds differ between Asian and non-Asian populations, and these may have a substantial influence on the development of cancer. To validate these findings, further research is required in these populations in the future.

## 5 Conclusions

This meta-analysis indicated that MASLD was associated with a higher risk of pancreatic cancer. To further strengthen the association, future prospective cohort studies should take into account different ethnic groups, diagnostic methods of fatty liver, the severity of MASLD, and potential confounding factors, as well as explore the potential mechanisms of pancreatic cancer development in MASLD patients. Clinicians should be alert of the chance of pancreatic cancer in these individuals and administer screening if necessary.

## Data availability statement

The original contributions presented in the study are included in the article/**Supplementary Material**. Further inquiries can be directed to the corresponding author.

## Author contributions

YZ: Conceptualization, Data curation, Formal analysis, Investigation, Methodology, Software, Supervision, Validation, Visualization, Writing – original draft, Writing – review & editing. B-GZ: Conceptualization, Data curation, Formal analysis, Methodology, Software, Visualization, Writing – original draft. J-DZ: Conceptualization, Formal analysis, Supervision, Writing – review & editing. B-BD: Investigation, Project administration, Supervision, Writing – original draft, Writing – review & editing.
