# Supplementary material for: Association between metabolic dysfunction-associated steatotic liver disease and risk of incident pancreatic cancer: a systematic review and meta-analysis of cohort studies
Source: Front Oncol. 2024 Mar 19;14:1366195. doi: 10.3389/fonc.2024.1366195 (PMC10985331; doi:10.3389/fonc.2024.1366195)

Supplementary online material

Association between metabolic dysfunction-associated steatotic liver disease and risk of incident pancreatic cancer: a systematic review and meta-analysis of cohort studies

Table S1. Description of excluded studies

Table S2. The other characteristics of included studies

Table S3. Methodological quality assessment of included studies

Figure S1. The Begg's funnel plot for overall meta-analysis

Table S1. Description of excluded studies			
**No.**	**First author**	**Publication year**	**Reason for exclusion**
1.	Sorensen	2003	Studies without relevant data
2.	Kato	2009	Conference abstract
3.	Ono	2015	Editorial
4.	Sanna	2016	Review
5.	Chang	2018	Case-control study
6.	Khoudari	2020	Conference abstract
7.	Lirendra	2020	Review
8.	Liu	2020	Meta-analysis
9.	Sbeit	2020	Case-control study
10.	Albhaisi	2021	Review
11.	Cuciureanu	2021	Review
12.	de Rezende	2021	Cross-sectional study
13.	Sbeit	2021	Studies without relevant data
14.	Sbeit	2021	Studies without relevant data
15.	Venniyoor	2021	Review
16.	Yamamoto	2021	Studies without relevant data
17.	Gumussoy	2022	Studies without relevant data
18.	Mantovani	2022	Meta-analysis
19.	McHenry	2022	Inappropriate confirmation method of NAFLD
20.	Afzaal	2023	Conference abstract
21.	Chan	2023	Meta-analysis
22.	Chowdary	2023	Conference abstract
23.	Ibrahim	2023	Review
24.	Singh	2023	Conference abstract
25.	Souza	2023	Meta-analysis
26.	Park	2022	Research on overlapping populations

27.	Park	2023	Research on overlapping populations

References for the table

1. Sorensen HT, Mellemkjær L, Jepsen P, et al. Risk of cancer in patients hospitalized with fatty liver -: A Danish cohort study. Journal of Clinical Gastroenterology. 2003;36(4):356-359.

2. Kato H, Isaji S, Murata Y, et al. Pancreatic cancer is a significant risk factor in the development of NAFLD/NASH after pancreaticoduodenectomy: Impact of high-dose pancreatic enzyme supplementation. Pancreas. 2009;38(8):1013.

3. Ono M. Is non-alcoholic fatty liver disease a systemic disease that promotes extrahepatic cancer development? Hepatology Research. 2015;45(7):711-713.

4. Sanna C, Rosso C, Marietti M, Bugianesi E. Non-Alcoholic Fatty Liver Disease and Extra-Hepatic Cancers. Int J Mol Sci. 2016;17(5):717.

5. Chang CF, Tseng YC, Huang HH, Shih YL, Hsieh TY, Lin HH. Exploring the relationship between nonalcoholic fatty liver disease and pancreatic cancer by computed tomographic survey. Intern Emerg Med. 2018;13(2):191-197.

6. Khoudari G, Eltelbany A, Sarmini MT, McCullough A. The Prevalence of Gastrointestinal Tract (GIT) and Non-GIT Cancers in Non-Alcoholic Fatty Liver in the USA: A Population-Based Study. American Journal of Gastroenterology. 2020;115(SUPPL):S507-S508.

7. Lirendra M, Simanihuruk V, Raharjo M, Permana SL, Purnama W, Lesmana CRA. Association between non-alcoholic fatty liver disease and extrahepatic malignancy risk and mortality: A systematic review. Hepatology International. 2020;14:S356.

8. Liu SS, Ma XF, Zhao J, et al. Association between nonalcoholic fatty liver disease and extrahepatic cancers: a systematic review and meta-analysis. Lipids Health Dis. 2020;19(1):118.

9. Sbeit W, Greener T, Kadah A, et al. Pancreato-biliary manifestations of non-alcoholic fatty liver disease: A case-control multi-center study. United European Gastroenterology Journal. 2020;8(8 SUPPL):846.

10. Albhaisi S, Sanyal AJ. Does Nonalcoholic Fatty Liver Disease Increase the Risk for Extrahepatic Malignancies? Clinical Liver Disease. 2021;17(3):215-219.

11. Cuciureanu T, Stanciu C, Zenovia S, et al. Non-alcoholic fatty liver disease and extra-hepatic malignancies. Journal of BUON. 2021;26(6):2411-2414.

12. de Rezende AQM, Gestic MA, Utrini MP, et al. Is there a link between non-alcoholic fatty liver disease aspects and pancreatic cancer? Results of a case-matched study. Revista do Colegio Brasileiro de Cirurgioes. 2021;48.

13. Sbeit W, Greener T, Kadah A, et al. Pancreatobiliary manifestations of nonalcoholic fatty liver disease: a retrospective case-control multicenter study. Eur J Gastroenterol Hepatol. 2021;33(5):722-726.

14. Sbeit W, Greener T, Kadah A, et al. Pancreatic and hepatobiliary manifestations of nonalcoholic fatty pancreatic disease: a referral multi-center experience. Eur J Gastroenterol Hepatol. 2021;33(1S Suppl

1):e297-e301.

15. Venniyoor A, Al Farsi AA, Al Bahrani B. The Troubling Link Between Non-alcoholic Fatty Liver Disease (NAFLD) and Extrahepatic Cancers (EHC). Cureus. 2021;13(8):e17320.

16. Yamamoto K, Ikeya T, Okuyama S, Fukuda K, Kobayashi D. The association between non-alcoholic fatty liver disease (with or without metabolic syndrome) and extrahepatic cancer development. J Gastroenterol Hepatol. 2021;36(7):1971-1978.

17. Gumussoy M, Koc O, Karatas G, et al. Factors associated with the development of extrahepatic malignancy in patients with nonalcoholic fatty liver disease: A single-center longitudinal study. European Journal of Gastroenterology and Hepatology. 2022;34(11):1172-1177.

18. Mantovani A, Petracca G, Beatrice G, et al. Non-alcoholic fatty liver disease and increased risk of incident extrahepatic cancers: a meta-analysis of observational cohort studies. Gut. 2022;71(4):778-788

19. McHenry S, Zong X, Shi M, et al. Risk of nonalcoholic fatty liver disease and associations with gastrointestinal cancers. Hepatol Commun. 2022;6(12):3299-3310.

20. Afzaal T, Hudson D, Vaan B, Khan MQ, Qumosani K, Teriaky A. Gastrointestinal malignancies in hospitalized patients with non-alcoholic fatty liver disease (NAFLD): Analysis of the national inpatient sample (NIS). Canadian Liver Journal. 2023;6(1):179-180.

21. Chan KE, Ong EYH, Chung CH, et al. Longitudinal Outcomes Associated with Metabolic Dysfunction-Associated Steatotic Liver Disease. A Meta Analysis of 129 Studies. Clin Gastroenterol Hepatol

2023 Sep 28:S1542-3565(23)00754-1. doi: 10.1016/j.cgh.2023.09.018. Online ahead of print.

22. Chowdary SB, Yadukumar L, Asija U, Sharma AM, Sharma N. Does non-alcoholic fatty liver disease (NAFLD) influence outcomes of pancreatic cancer (PANC)? A nationwide analysis. Journal of Clinical Oncology. 2023;41(16).

23. Ibrahim MK, Simon TG, Rinella ME. Extrahepatic Outcomes of Nonalcoholic Fatty Liver Disease: Nonhepatocellular Cancers. Clinics in Liver Disease. 2023;27(2):251-273.

24. Singh RR, Chawla S. NON-ALCOHOLIC FATTY LIVER DISEASE IS AN INDEPENDENT RISK FACTOR FOR PANCREATICOBILIARY CANCERS - A POPULATION-BASED STUDY. Gastroenterology. 2023;164(6):S-1311.

25. Souza M, Diaz I, Barchetta I, Mantovani A. Gastrointestinal cancers in lean individuals with non-alcoholic fatty liver disease: A systematic review and meta-analysis. Liver Int. 2023 Oct 13. doi: 10.1111/liv.15763. Epub ahead of print.

26. Park JH, Hong JY, Han K, Kang W, Park JK. Increased risk of pancreatic cancer in individuals with non-alcoholic fatty liver disease. Sci Rep. 2022;12(1):10681.

27. Park JH, Hong JY, Shen JJ, Han K, Park JO, Park YS, et al. Increased Risk of Young-Onset Digestive Tract Cancers Among Young Adults Age 20-39 Years With Nonalcoholic Fatty Liver Disease: A Nationwide Cohort Study. J Clin Oncol. 2023;41(18):3363-3373.

Table S2. The other characteristics of included studies			
**First author (year)**	**Study areas**	**Mean age (years)**	**Adjusted confounding factors**
Sun (2015)	Asia	61	Age, sex, obesity, hypertension, diabetes, dyslipidemia, viral hepatitis, colon polyps, diagnostic procedures of esophagogastroduodenoscopy, colonoscopy, abdomen computed tomography, or ultrasonography
Kim (2018)	Asia	48	Age, sex, smoking, hypertension, diabetes, lipids, and gamma-glutamyltransferase levels
Allen (2019)	North America	54	Age, sex, and obesity
Simon (2021)	Europe	51	Age at the index date, sex, calendar year, county of residence, cardiovascular disease, diabetes, hypertension, dyslipidemia, obesity, endstage renal disease, family history of cancer at age <50 years, education (three groups plus missing category), the number of recorded hospital encounters in the year preceding the index biopsy date (or corresponding matching date), and alcohol abuse/misuse
Wang (2021)	Asia	53	Age, physical activity, smoking status, body mass index, diabetes, hypertension, alcohol intake, education level, serum levels of alanine aminotransferase, high-density lipoprotein cholesterol, total cholesterol, triglycerides, and C-reactive protein
Liu (2022)	Europe	58	Age at enrollment, sex, education, assessment center, household income, smoking status, pure alcohol intake, and physical activity
Chung (2023)	Asia	46	Age, sex, income, smoking, exercise, estimated glomerular filtration rate, Charlson comorbidity index score, waist circumference, glucose, total cholesterol and systolic blood pressure
Wei (2023)	Asia	53	Age, sex, smoking status, and alcohol status
Yuan (2023)	Asia	50	Age, sex, education level, smoking status, alcohol consumption, physical activity, and family history of cancers

Table S3. Methodological quality assessment of included studies					
**First author (Year)**	**Selection**	**Comparability**	**Outcome**	**Total (0-9)**	**Quality**
Sun (2015)	☆☆☆☆	☆☆	☆☆	8	Moderate
Kim (2018)	☆☆☆	☆☆	☆☆	7	Moderate
Allen (2019)	☆☆☆	☆	☆☆☆	7	Moderate
Simon (2021)	☆☆☆☆	☆☆	☆☆☆	9	High
Wang (2021)	☆☆☆☆	☆	☆☆☆	8	Moderate
Liu (2022)	☆☆☆☆	☆☆	☆☆☆	9	High
Chung (2023)	☆☆☆☆	☆☆	☆☆☆	9	High
Wei (2023)	☆☆☆☆	☆☆	☆☆	8	Moderate
Yuan (2023)	☆☆☆☆	☆☆	☆☆☆	9	High
Note: NOS, Newcastle–Ottawa Scale.					

Figure S1. The Begg's funnel plot for overall meta-analysis

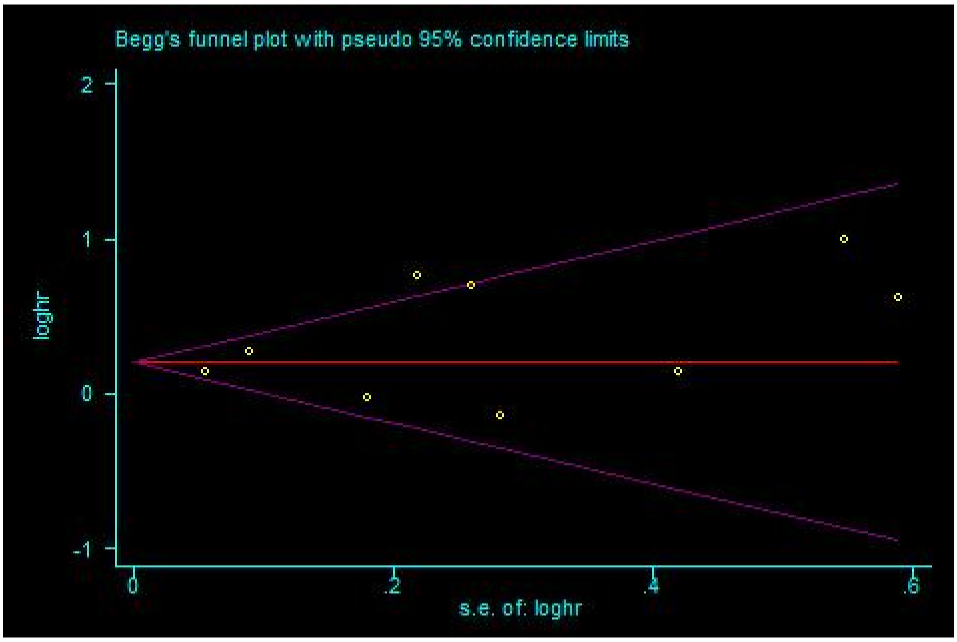

Supplement: Supplementary file 1 [file DataSheet_1.pdf]
